# Characterisation of tuberculosis mortality in informal settlements in Nairobi, Kenya: analysis of data between 2002 and 2016

**DOI:** 10.1186/s12879-021-06464-2

**Published:** 2021-07-31

**Authors:** Judy Gichuki, Donnie Mategula

**Affiliations:** 1 Nairobi City County Government, Health Services Department, P.O. Box 34349, Nairobi, 00100 Kenya; 2 Malawi-Liverpool-Wellcome Trust Clinical Research Programme, P.O Box 30096, Blantyre, Malawi

**Keywords:** Tuberculosis, Verbal autopsy, Nairobi, Informal settlements

## Abstract

**Background:**

Tuberculosis (TB) remains one of the key public health problems in Africa. Due to multifaceted challenges, its burden is poorly described in informal settlements. We describe tuberculosis mortality in two informal settlements in Nairobi, Kenya.

**Methods:**

This is a secondary analysis of 2002–2016 verbal autopsy data from informal settlements in the Nairobi Urban Health Demographic Surveillance System (NUHDSS). A descriptive analysis of deaths assigned as caused by TB was done. Pearson chi-square tests were used to determine differences between socio-demographic factors. Logistic regression was carried out to examine the risk of death from TB within the characteristics.

**Results:**

There were 6218 deaths in the NUHDSS within the period of analysis, of which 930 (14.96%) were deaths from TB. The average number of TB deaths per year was 62(SD 23.9). There was a reduction in TB deaths from 21.2% in 2005 to 1.7% in 2016. Males had 1.39 higher odds of dying from TB than females (AOR 1.39; 95% CI 1.18–1.64; *p*-value < 0.001). Compared to those aged 30–39 years, the ≥50-year-olds had a 42% lower chance of dying from TB (AOR 0.57; 95% CI 0.47–0.73; *p*-value < 0.001). Those dying at home had 1.39 odds of dying from TB as compared to those who died in a health facility(AOR 1.93; 95% CI 1.17–1.64; *p* value< 0.001).

**Conclusion:**

There was a reduction in TB deaths over the study period. Males had the highest risk of death. There is a need to strengthen TB surveillance and access to TB diagnosis and treatment within informal settlements to enhance early diagnosis and treatment.

**Supplementary Information:**

The online version contains supplementary material available at 10.1186/s12879-021-06464-2.

## Introduction

Tuberculosis (TB) ranks as one of the top causes of morbidity and mortality globally. The global incidence of TB is estimated at an average of 130 cases per 100,000 population per year with approximately 10 million people being infected with TB in 2018 [1]. The 2019 global TB report estimates that there were approximately 1.5 million people that died of TB in 2018. Sub-Saharan Africa bears the highest global TB burden and over 50% of TB cases in the region are co-infected with HIV [2]. As part of the goal to end the global TB pandemic, the World health organization(WHO) has set targets aimed at a 90% reduction in global TB deaths and 80% reduction in the global incidence of TB by the year 2030 from the baseline rates of the year 2015 [3].

Kenya is ranked among the high TB burden countries. As of 2017, the estimated TB incidence was 319 cases per 100, 000 population while TB mortality was estimated at 50/100, 000 [4]. The 2016 Kenya TB survey found a TB prevalence of 558 per 100,000 people. Males and those living in urban settlements were found to have a higher burden of the disease. There were 2.5 times higher TB cases in males as compared to females while urban areas had a 1.7 higher TB prevalence as compared to rural areas [5]. It is estimated that over 40% of TB cases are undetected at the health facility level hence affirming the need for data triangulation to improve TB surveillance [5].

TB mortality surveillance is important in assessing programmatic performance [6]. There are several methods of determining TB attributable deaths which include: direct estimation from national vital registration systems, estimation using verbal autopsy data and indirect estimation using TB patient cohort case-fatality data [7]. In Kenya, vital registration systems are not well established to detect and report all TB related deaths, as, a majority of the deaths do not undergo medical certification [8]. Estimating TB burden from treatment outcome data is often biased due to incomplete reports, loss to follow up, patient transfer outs and undetected recurrent infections [6]. Analysis of verbal autopsy(VA) data within demographic surveillance sites plays a crucial role in estimating TB related deaths and in TB mortality surveillance and is useful in the triangulation of facility-based data as well as vital registration data on TB related mortality [7].

Defining the burden of disease in informal settlements is often difficult as these populations have multiple access barriers (financial, physical, social or cultural).VA data assists in describing specific community-based mortality trends in such informal settlements [8]. This paper utilizes the Nairobi Urban Health Demographic Surveillance System (NUHDSS) cause of death data to assess TB mortality in the urban informal settlements of Nairobi. This is important as those residing in informal settlements are more prone to TB deaths with some studies finding up to five times higher risk of TB mortality for those from lower socioeconomic backgrounds [9].

## Methods

### Study area

The TB mortality data used in this paper was collected from two informal settlements (Korogocho and Viwandani) in Nairobi Kenya, that form the NUHDSS, a demographic surveillance system run by the African population health research centre(APHRC). Korogocho is a large slum in Nairobi, Kenya. It is located in Ruaraka Sub-County in Nairobi and covers an area of 0.9 square km with a total population of 36,900 and a density of 42,401 persons per km^2^ as per the 2019 Kenya population and housing census [10]. Viwandani is located in Makadara Sub-County, it covers an area of 5 km^2^ with a population of 43,070 and a density of 8554 persons per square km [10]. A map for the study area can be accessed in Additional file 1.

### Data

The NUHDSS VA dataset consisted of 1) responses to a questionnaire that captured events surrounding the death from the deceased person’s close relative(s) or caregiver(s) who was aware of the circumstances surrounding the death 2) the physicians’ interpretation of likely cause of death from the deceased signs and symptoms following the VA and 3) the output from the InterVA-4 software that consists of up to 3 likelihoods attributed to each death. The InterVA-4 software uses probabilistic models based on Bayes’ theorem to interpret symptom data and determine possible causes of death [11] and has been validated in similar settings [12].

### Data analysis

We used STATA 15 for the analysis. We performed a descriptive analysis of deaths assigned as caused by TB within the NUHDSS from 2002 to 2016. We determined the trend of the deaths over the 15 years of analysis. TB deaths were characterized based on socio-demographic characteristics of age, sex, social health-seeking patterns etc. and summarized in tables and graphs. Given the documented differences in TB prevalence and morbidity by sex [5], Pearson-Chi-square tests were used to determine if health care seeking, place of death and age characteristics were statistically different based on sex. Adjusted logistic regression analysis was carried out to examine the risk of death from TB within the socio-demographic characteristics (age, sex, place of death, year of death, slum area in NUHDSS and whether healthcare was sought for the illness). In the logistic regression analysis, the dependent variable was derived from the entire NUHDSS mortality data and consisted of a binary variable defining whether one died from TB or not. A backward approach was used to build the model, retaining all the variables unless they were collinear with other variables in the model.

## Results

**Fig. 1:**
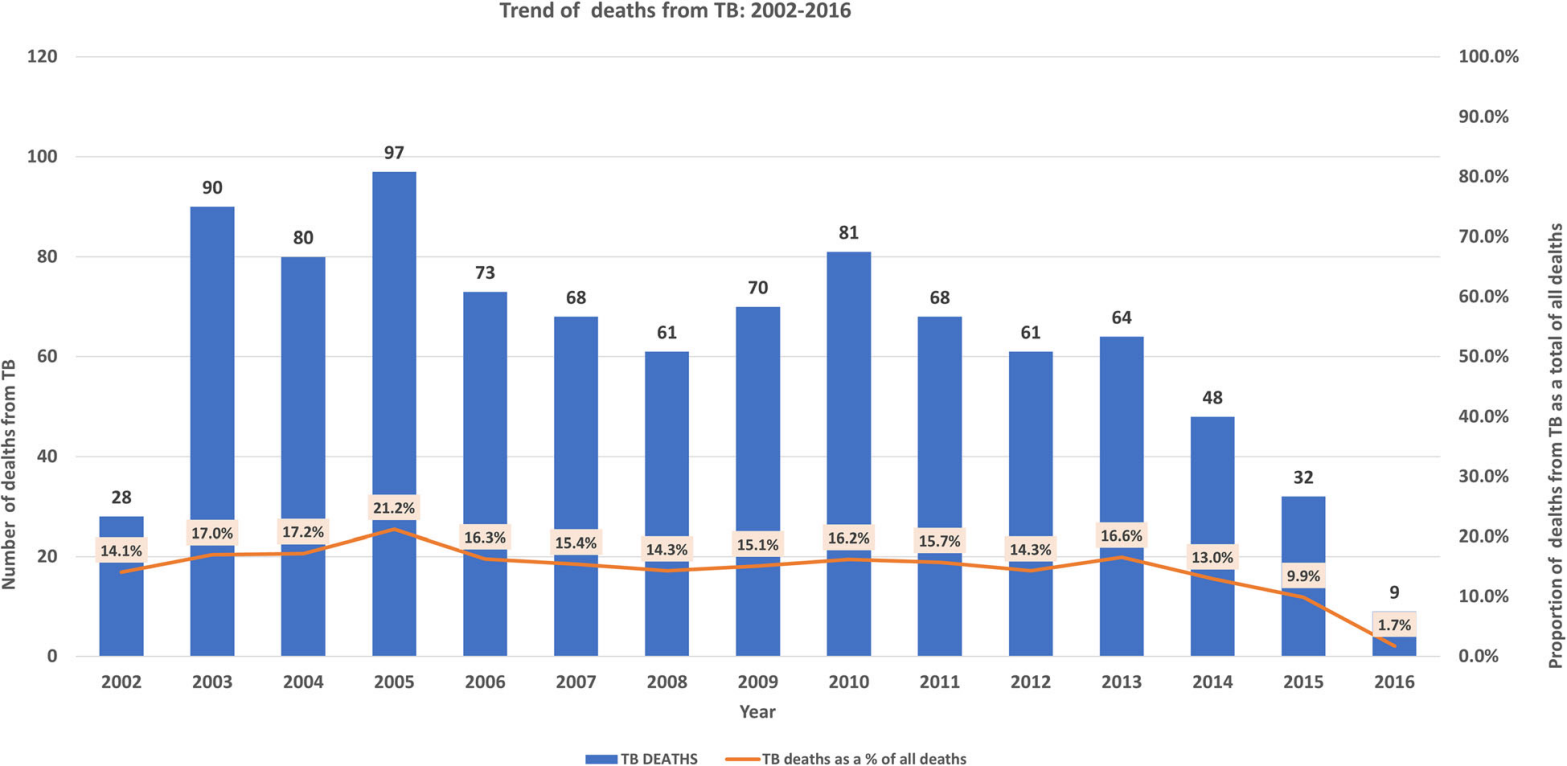
Trend of deaths from TB in the NUHDSS: 2002-2016

There were 6218 deaths in the NUHDSS from 2002 to 2016, of which 930 (14.96%) were assigned as deaths from TB. The average number of deaths from TB per year was 62 (SD 23.9) with the highest being in 2005 (21.2% *n* = 97). Figure 1 shows the trend of deaths from TB in the NUHDSS. Other causes of death in the NUHDSS have been published elsewhere [13].

The graph shows the percentage of deaths from TB as a total of all deaths in the NUHDSS. There was a steady increase from 2002 (14.1%) to 2005 (21.2%), followed by a steady decline from 2005 (21.2%) to 2016 (1.7%) with the sharpest downward trend from 2014 (13%) to 2016 (1.7%).

### Socio-demographic characteristics

Majority of deaths from TB were in the 30 to 39 age categories (*n* = 314, 33.8%) followed by the 40–49 age category (*n* = 222, 23.9%). The lowest number of deaths from TB were in the 0–9 years category (*n* = 19, 2%). There was a 14.4% higher proportion of TB deaths among males as compared to females (57.2% vs 42.8%).

The deaths from TB in Korogocho (*n* = 580,62.4%) were nearly twice those in Viwandani (*n* = 350,37.6%). A majority(*n* = 475,51.1%) of the TB deaths happened at the health facility followed by those that happened in the house of the deceased (*n* = 396, 42.6%), however, death certificates were only issued and seen in 1.2% of the TB deaths (Table 1).

**Table 1:** Socio-demographic characteristics of the people that died from TB

Variable	Count (***N*** = 930)	Percentage
**Age (Years)**	*n* = 930	
0–9	19	2.0
10–19	24	2.6
20–29	164	17.6
30–39	314	33.8
40–49	222	23.9
≥ 50	187	20.1
**Sex**	*n* = 930	
Female	398	42.8
Male	532	57.2
**Area in the NUHDSS**	*n* = 930	
Korogocho	580	62.4
Viwandani	350	37.6
**Place of death**	*n* = 929	
House	396	42.6
Health facility	475	51.1
Enroute to health facility	40	4.3
Other	18	1.9
**Death certificate issued**	*n* = 927	
No	624	67.3
Yes – seen records	11	1.2
Yes – Not seen records	85	9.2
Don’t know (Unsure)	207	22.3
**Sought healthcare for illness**	*N* = 929	
Yes	902	97.0
No	19	2.0
Don’t know	8	0.9

### Health care seeking characteristics

**Fig. 2:**
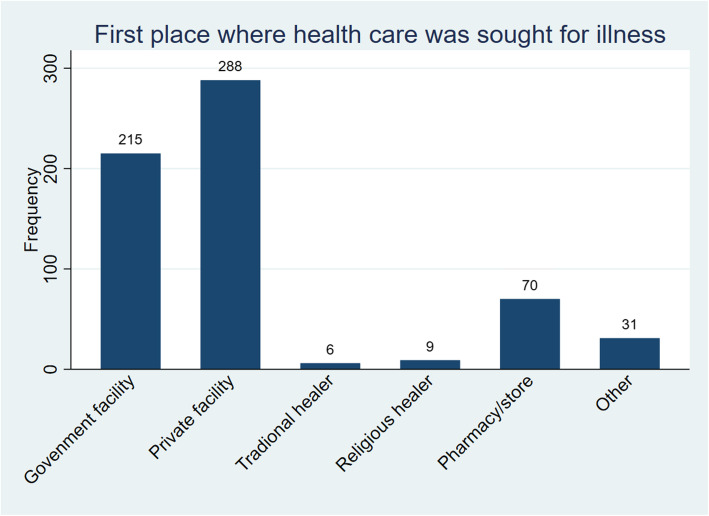
First place where health care was sought for illness

A total of 902 (97%) of those who died from TB sought health care for the illness before the death. A majority first sought health care from a private facility (*n* = 288, 31.9%) while 70 (7.8%) first visited a pharmacy or store. A few of the deceased first sought health care from a traditional healer (*n* = 6,0.7%) while some visited religious healers (*n* = 9, 1%). Data for where healthcare was first sought was missing for 283 (31.4%) of the deceased though it was reported that they sought healthcare (Fig. 2).

### Associations between sex and other socio-demographic characteristics

Table 2 shows the associations between sex and the other social demographic characteristics of the people that died of TB in the NUHDSS. Health care-seeking behaviour among those that died of TB varied based on sex (*p* value = 0.017), 99% of females (*n* = 394) were reported to have sought health care as opposed to 95.6% of the males (*n* = 508). There was also strong evidence that the place of death varied by sex (*p* = 0.015). A higher percentage of males died in the house (46.1%) as compared to females (37.9%), while a higher percentage of females died in a hospital (57%) as compared to males (46.6%). There was a statistically significant association between the age category and sex of those who died from TB (*p* value< 0.001). A higher percentage of females in the 20–29 years age range (27.4%) died from TB as compared to males within the same age group (10.3%).

**Table 2:** Associations between sex and other socio-demographic characteristics

	Female	Male	Pearson Chi^**2**^ ***P***-Value
**Sought healthcare for illness/injury**			
Yes	394 (99%)	508 (95.6%)	0.017
No	2 (0.5%)	17 (3.2%)	
Don’t Know	2 (0.5%)	6 (1.1%)	
Total	398	531	
**Place of death**			
House	151 (37.9%)	245 (46.1%)	0.015
Health facility	227 (57.0%)	248 (46.6%)	
Enroute to health facility	16 (4.0%)	24 (4.5%)	
Other	4 (1%)	14 (2.6%)	
Total	398	531	
**Age (Years)**			
0–9	9 (2.3%)	10 (1.9%	< 0.001
10–19	12 (3.0%)	12 (2.3%)	
20–29	109 (27.4%)	55 (10.3%)	
30–39	130 (32.7%)	184 (34.6%)	
40–49	82 (20.6%)	140 (26.3%)	
≥ 50	56 (14.1%)	131 (24.6%)	
Total	398	532	

Table 3 shows the adjusted logistic regression associations of risk of death from TB within various socio-demographic characteristics of the deceased in the NUHDSS. There was strong evidence that sex was associated with the risk of death from TB (*p*-value < 0.0001). Males had 1.39 higher odds of dying from TB than females (AOR 1.39; 95% CI 1.18–1.64; *p*-value < 0.001). Similarly, age was also associated with the risk of death from TB. Compared to those aged 30–39 years, those that were 50 years and above had a 43% lower chance of dying from TB (AOR 0.57; 95% CI 0.47–0.73; *p*-value < 0.001). Those that died between 2014 and 2016 had a 51% lower chance of dying from TB as compared to those that died between 2002 and 2005 (AOR 0.49; 95% CI 0.37–0.65; *p*-value < 0.001). Place of death was associated with risk of dying from TB with those dying at home having 1.39 odds of dying from TB as compared to those who died in a health facility (AOR 1.39; 95% CI 1.17–1.64; *p* value< 0.001).

**Table 3:** Adjusted logistic regression analysis to examine the risk of death from TB within various socio-demographic characteristics

Variables	AOR	95% CI		Wald ***P*** value	LR ***P*** value
		Lower	Upper		
Sex					
Female	Ref				*P* = 0.001
Male	1.39	1.18	1.64	< 0.001	
Age category (Years)					
0–9	0.02	0.01	0.03	< 0.001	*p* < 0.001
10–19	0.43	0.27	0.68	< 0.001	
20–29	0.73	0.58	0.92	0.007	
30–39	Ref				
40–49	1.02	0.82	1.27	0.863	
50 plus	0.58	0.47	0.73	< 0.001	
Slum area					
Korogocho	Ref				0.786
Viwandani	1.02	0.86	1.20	0.828	
Year of death categories					
2002–2005	Ref				*p* < 0.001
2006–2009	1.06	0.86	1.31	0.57	
2010–2013	0.90	0.73	1.10	0.289	
2014–2016	0.49	0.37	0.65	< 0.001	
Place of death					
Health facility	Ref				*p* < 0.001
House	1.39	1.17	1.64	< 0.001	
En route to health facility	1.23	0.83	1.83	0.297	
Other	0.51	0.29	0.90	0.02	
Missing	2.07	0.09	46.97	0.647	
Sought healthcare for illness/injury					
Yes	Ref				*p* < 0.001
No	0.06	0.04	0.10	< 0.001	
Don’t know	0.57	0.25	1.32	0.193	
Missing	0.17	0.01	3.53	0.254	

*CI* Confidence interval, *AOR* Adjusted odds ratio, adjusted for the variables in the table, *Ref* Reference group, *LR* Likelihood ratio

## Discussion

Like many other countries in the sub Saharan African region, Kenya is committed to reducing deaths due to tuberculosis in line with the WHO’s end TB strategy [3]. To achieve this goal, TB surveillance needs to be reliable to assist in appropriately directing resources, even in difficult to reach areas like informal settlements. The epidemiology of TB mortality in Nairobi informal settlements has evolved over the years as shown in this paper. This paper describes TB deaths that took place in the NUHDSS in Viwandani and Kochorogo in the years 2002 to 2016. The highest number of TB deaths for this period were in the age group 30 to 39 years. This is the same age group that had the highest HIV prevalence for the period of analysis [14, 15]. An HIV prevalence survey done in the same area in 2007 showed a 12% HIV prevalence in the two informal settlements, the highest prevalence being in the 30 to 39 age group [16].

Though HIV -TB co-infection data was not available for analysis, evidence shows that the risk of developing TB is estimated to be between 15 to 22 times greater in people living with HIV than among those without HIV infection [17].

There was a decline in TB deaths between the periods 2005 to 2016. Interventions targeting informal settlements in Nairobi across the years may have contributed to this decline. These include active defaulter tracing mechanisms from 2005, countrywide scale up and utilization of community health volunteers for home-based care follow-ups and community-based direct observed treatment short course (DOTS) from 2007 and creation of support groups for HIV/TB co-infected patients [18–20].

There was a higher proportion of TB deaths in males as compared to females in the NUHDSS within the study period. The Kenya 2016 TB prevalence survey found a similar pattern of higher TB cases in males in comparison to females [5]. Contributing factors may include poorer health-seeking behaviour among men as compared to women [21] and higher TB risk factors among men such as smoking, alcohol and occupational exposure to undetected TB cases [22]. The risk of TB disease has been found to increase by 3.3 and 1.6 times respectively as a result of alcohol use disorder and tobacco smoking [23]. Poorer health-seeking behaviour was also noted in the NUHDSS with a higher proportion of females being reported to have sought health care for their illness as compared to males. Similarly, in a study in Zambia, males were less likely to seek care for their presumptive TB symptoms [21].

Findings in this study demonstrate the crucial role of care-seeking in morbidity and mortality patterns. Nearly 43% of the deaths from TB happened at home. The immediate cause of death in most pulmonary TB patients is usually septic shock or respiratory emergencies [24] and these would require urgent hospital care. Though access to care data was not available for analysis, the high number of deaths at home could be an indicator of access to care challenges. People in informal settlements such as Korogocho and Viwandani often have health care access challenges [25], explaining why a proportion of the patients die at home.

Health-seeking behaviour may also have been affected by the low perception of the seriousness of symptoms, stigma for TB related symptoms because of its correlation with HIV, and delayed care-seeking due to poor awareness of the cardinal signs and symptoms of TB [26]. Community awareness on TB signs and symptoms and subsequent follow-up measures have a crucial role in enhancing appropriate and timely care seeking [27]. Though the informal settlements in Nairobi are supported by a strong community health strategy that consists of a network of community health volunteers (CHVs) organized in community health units (CHUs) [28], referral linkages between the CHVs and health facilities are often weak due to incomplete referrals, inadequate documentation tools as well as poor counter referral mechanisms [27]. This makes it difficult for CHVs to track back the referrals made and whether care was received. Improving the CHU linkages with health facilities may therefore have a major role in enhancing early and effective referral of presumptive TB patients.

Furthermore, the high number of deaths at home (43%) and the few numbers of deaths that were reported to have had death certificates issued (9.1%), illustrate the key role that verbal autopsies can play in establishing the cause of death statistics within the urban informal settlements. In Kenya, deaths at home or in the community do not ordinarily undergo medical certification. They are usually registered by the assigned local registration agent (usually the assistant chief) who is only required to identify the most probable cause of death from a list in the death registration form [29].

Health care-seeking patterns for TB symptoms in the two NUHDSS areas were from multiple sources with the majority having sort for care first in a private health facility. TB service diagnosis and treatment availability in most private health facilities are limited, leading to multiple hospital visits before a diagnosis is made [27]. According to the 2017 patient pathway analysis for differentiated service delivery of TB in Kenya, only 46% of people with possible TB who sought health care had access to diagnosis at initial care seeking [30]). Findings in our study, therefore, re-emphasize the need for strengthening of the capacity of the private health care providers in TB knowledge, diagnosis and treatment especially within the informal settlements as per the Kenya Public-Private Mix (PPM) 2017–2020 action plan and the Kenya national strategic plan for tuberculosis, leprosy and lung health 2019–2023. The multiple type of facilities where care was sought from before death, reinforces the role of the PPM approach in reaching out to a wider scope of facilities including pharmacies, small private clinics, nursing homes and stand-alone laboratories that have ordinarily been left out within the TB scope of service to detect TB cases and in making appropriate referrals for diagnosis and treatment [31]. This would lead to improved detection rates, treatment outcomes, enhance access to services and subsequently minimize late case detection and resultant TB mortalities [26].

Some limitations for this paper are that VA data analysis is usually dependent on the review of symptoms generally associated with various diseases or conditions and VA data may under/overestimate TB related deaths [30]. Other challenges are that mortality estimates obtained by VA are susceptible to bias due to misclassification [7]. Even with these limitations, the dataset used in the analysis covered a large population over a long period and expounds on the community patterns of TB mortality within the informal settlements of Nairobi.

## Conclusion

In conclusion, this study finds that there was a reduction in TB deaths over the study period in the NUHDSS. There were notable sex differences in TB mortality in the two informal settlements with males nearly 40% more likely to die of TB as compared to females. Over four out of ten of the TB deaths occurred at home. There is a need to strengthen community awareness, TB surveillance and access to TB diagnosis and treatment especially in private facilities within informal settlements to enhance early diagnosis and treatment.

## Supplementary Information

**Additional file 1.**

## Data Availability

The KENYA - NUHDSS - Verbal Autopsy, causes of deaths 2002–2015 dataset was used in this secondary analysis. The dataset is available upon request from the APHRC Microdata Portal http://microdataportal.aphrc.org/index.php/catalog. Access to the dataset can be obtained through submission of a written request in the APHRC portal following creation of an account. Further information on how to apply for access to the data can be found here (http://microdataportal.aphrc.org/index.php/how-to-use-it).

## Abbreviations

AOR: Adjusted Odds Ratio
APHRC: African population health research centre
CHUs: Community Health Units
CHVs: Community Health Volunteers
CI: Confidence interval
DOTS: Direct-Observed Treatment Short course
DOTS: Direct-Observed Treatment Short course
HIV: Human Immunodeficiency Virus
NUHDSS: Nairobi Urban Health Demographic Surveillance System
PPM: Public Private Mix
SD: Standard deviation
TB: Tuberculosis
VA: Verbal Autopsy
WHO: World Health Organisation

## References

[1] World Health Organization (2020). The Global Tuberculosis Report 2019.

[2] Zumla A, Petersen E, Nyirenda T, Chakaya J (2015). Tackling the tuberculosis epidemic in sub-Saharan Africa - unique opportunities arising from the second European developing countries clinical trials partnership (EDCTP) programme 2015-2024. Int J Infect Dis.

[3] World Health Organization, World Health Organization. WHO calls on countries and partners to "Unite to End Tuberculosis". 2016. Available from https://www.who.int/news/item/22-03-2016-who-calls-on-countries-and-partners-to-unite-to-end-tuberculosis-.

[4] Centre for Disease Control(CDC) (2020). Kenya Tuberculosis Country Profile.

[5] Kenya Ministry of Health. Kenya Tuberculosis Prevalence Survey. 2016;(August):43–47. Available from: https://www.researchgate.net/publication/329926671_Kenya_tuberculosis_prevalence_survey_2016_Challenges_and_opportunities_of_ending_TB_in_Kenya

[6] Bhargava A, Bhargava M (2020). Tuberculosis deaths are predictable and preventable: Comprehensive assessment and clinical care is the key. J Clin Tuberc Other Mycobact Dis.

[7] Korenromp EL, Bierrenbach AL, Williams BG, Dye C. The measurement and estimation of tuberculosis mortality. Int J Tuberc Lung Dis. 2009;13(3):283–303. Available from: https://pubmed.ncbi.nlm.nih.gov/19275787/. 19275787

[8] Measure Evaluation (2013). National civil registration and vital statistics system: baseline systems assessment report.

[9] Arcoverde MAM, Berra TZ, Alves LS, Dos Santos DT, Belchior ADS, Ramos ACV, Arroyo LH, De Assis IS, Alves JD, De Queiroz AAR, Yamamura M, Palha PF, Neto FC, Silva-Sobrinho RA, Nihei OK, Arcêncio RA (2018). How do social-economic differences in urban areas affect tuberculosis mortality in a city in the tri-border region of Brazil, Paraguay and Argentina. BMC Public Health.

[10] Kenya National Bureau of Statistics (2019). 2019 Kenya Population and Housing Census: Volume II.

[11] Byass P, Fottrell E, Huong DL, Berhane Y, Corrah T, Kahn K, Muhe L, Duc Van D (2006). Refining a probabilistic model for interpreting verbal autopsy data. Scand J Public Health.

[12] Bauni E, Ndila C, Mochamah G, Nyutu G, Matata L, Ondieki C, Mambo B, Mutinda M, Tsofa B, Maitha E, Etyang A, Williams TN (2011). Validating physician-certified verbal autopsy and probabilistic modeling (InterVA) approaches to verbal autopsy interpretation using hospital causes of adult deaths. Popul Health Metr.

[13] Mategula D, Gichuki J. Does recall time matter in verbal autopsies? Evidence from urban informal settlements in Nairobi, Kenya. Wellcome Open Res. 2021;5 Available from: https://pubmed.ncbi.nlm.nih.gov/33869793/. [cited 2021 Jun 15]. 10.12688/wellcomeopenres.16243.1 PMC8030111 33869793

[14] Kimanga DO, Ogola S, Umuro M, Ng’ang’a A, Kimondo L, Murithi P, Muttunga J, Waruiru W, Mohammed I, Sharrif S, De Cock KM, Kim AA (2014). Prevalence and incidence of HIV infection, trends, and risk factors among persons aged 15-64 years in Kenya: results from a nationally representative study. J Acquir Immune Defic Syndr.

[15] Kenya National AIDS and STI Control Programme (2007). Kenya AIDS Indicator Survey 2007.

[16] Madise NJ, Ziraba AK, Inungu J, Khamadi SA, Ezeh A, Zulu EM, et al. Are slum dwellers at heightened risk of HIV infection than other urban residents? Evidence from population-based HIV prevalence surveys in Kenya. Health Place. 2012;18(5):1144–52. Available from: https://pubmed.ncbi.nlm.nih.gov/22591621/. 10.1016/j.healthplace.2012.04.003 PMC3427858 22591621

[17] World Health Organization, World Health Organization. Global tuberculosis report 2015. 2015. Available from: https://www.who.int/tb/publications/global_report/gtbr15_main_text.pdf.

[18] Malteser International (2016). Fighting AIDS and TB in the slums of Nairobi.

[19] Thomson KA, Cheti EO, Reid T (2011). Implementation and outcomes of an active defaulter tracing system for HIV, prevention of mother to child transmission of HIV (PMTCT), and TB patients in Kibera, Nairobi, Kenya. Trans R Soc Trop Med Hyg.

[20] WHO (2009). A brief history of tuberculosis control in Kenya.

[21] Chanda-Kapata P, Kapata N, Masiye F, Maboshe M, Klinkenberg E, Cobelens F, et al. Health seeking behaviour among individuals with presumptive tuberculosis in Zambia. PLoS One. 2016;11(10) Available from: https://pubmed.ncbi.nlm.nih.gov/27711170/. [cited 2021 Mar 21]. 10.1371/journal.pone.0163975 PMC5053535 27711170

[22] Helfinstein S, Engl E, Thomas BE, Natarajan G, Prakash P, Jain M, Lavanya J, Jagadeesan M, Chang R, Mangono T, Kemp H, Mannan S, Dabas H, Charles GK, Sgaier SK. Understanding why at-risk population segments do not seek care for tuberculosis: A precision public health approach in South India. BMJ Glob Heal. 2020 ;5(9). Available from: https://pubmed.ncbi.nlm.nih.gov/32912854/. [cited 2021 Mar 21] 10.1136/bmjgh-2020-002555 PMC7482470 32912854

[23] World Health Organization (2020). Tuberculosis key facts 2020.

[24] Lin CH, Lin CJ, Kuo YW, Wang JY, Hsu CL, Chen JM, Cheng WC, Lee LN (2014). Tuberculosis mortality: patient characteristics and causes. BMC Infect Dis.

[25] Wamukoya M, Kadengye DT, Iddi S, Chikozho C (2020). The Nairobi urban health and demographic surveillance of slum dwellers, 2002–2019: value, processes, and challenges. Glob Epidemiol.

[26] World Health Organisation. Working meeting: Public Private Mix (PPM) models for the sustainability of successful TB control initiatives: WHO; 2015. [cited 2021 Apr 8]; Available from: http://www.who.int/tb/areas-of-work/engaging-care-providers/public-private-mix/en/

[27] Kenya Ministry of Health (2019). National strategic plan for tuberculosis, leprosy and lung health 2019–2023.

[28] Paris-Saper M. Exploring community health through the lens of the community unit in Kariobangi north and the surrounding areas. Indep Study Proj Collect. 2150. 2015:1-31. Available from: https://digitalcollections.sit.edu/isp_collection/2150.

[29] Measure Evaluation (2013). Kenya National Civil Registration Vital Statistics:baseline systems assessment report.

[30] Masini E, Hanson C, Ogoro J, Brown J, Ngari F, Mingkwan P, Makayova J, Osberg M (2017). Using patient-pathway analysis to inform a differentiated program response to tuberculosis: the case of Kenya. J Infect Dis.

[31] AMREF (2020). Public-Private Mix (PPM) Interventions to Find the Missing People with TB in Kenya - Newsroom.

