# Supplementary figures and images for: Characterisation of tuberculosis mortality in informal settlements in Nairobi, Kenya: analysis of data between 2002 and 2016
Source: BMC Infect Dis. 2021 Jul 31;21:718. doi: 10.1186/s12879-021-06464-2 (PMC8325236; doi:10.1186/s12879-021-06464-2)

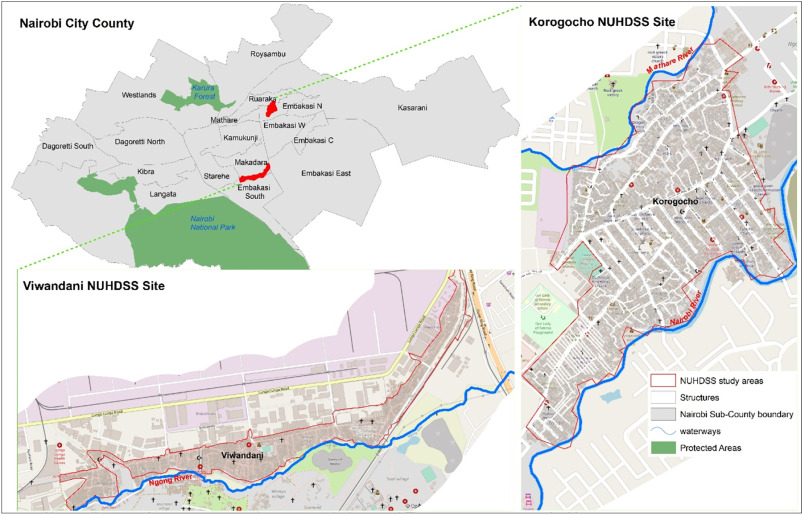

Supplement: Supplementary file 1 — Additional file 1. [file 12879_2021_6464_MOESM1_ESM.jpg]
